# In vitro fertilization with granulosa cell tumor: a report of two cases

**DOI:** 10.1007/s10815-018-1280-8

**Published:** 2018-08-09

**Authors:** Tiffany Phyoe-Battaglia, Chantal Bartels, John Nulsen, Daniel R. Grow

**Affiliations:** 1 St Francis Hospital, Hartford, CT USA; 2 University of Connecticut Health Center, Farmington, CT USA; 3 The Center for Advanced Reproductive Services, 2 Batterson Park Rd, Farmington, CT 06032 USA

## Background

Granulosa cell tumors (GCT), a subset of sex cord-stromal tumors, encompass 1–2% of all ovarian tumors and 2–5% of all ovarian malignancies [1]. The adult subtype is predominant, and molecular testing reveals a mutation in the FOXL2 gene in 97% of tumors examined [2]. There is increased incidence of granulosa cell tumors in non-white, obese women with family history of breast or ovarian cancer. Adult GCT is the most common ovarian tumor to produce endocrinopathy, and patients may present with an abdominal mass and symptoms of hyperestrogenism, irregular menses, abnormal uterine bleeding, post-menopausal bleeding, breast tenderness, and sexual precocity in children. Treatment involves complete resection and surgical staging followed by either observation for low-risk stage 1 cancer or platinum-based chemotherapy for higher stage cancer [3]. Initial fertility sparing surgery can be an option for patients but should be followed by total hysterectomy and bilateral salpingo-oophorectomy once childbearing is complete as recurrence can occur many years later [4].

AMH and inhibin B levels are found to be elevated and can be used as a tumor marker [5]. AMH elevations are proportional to tumor size and can range from normal reproductive age levels of < 5 ng/mL to levels greater than 100 ng/mL that far exceed normal [6]. AMH levels are now routinely used in the diagnosis and treatment of infertility, particularly to assess for ovarian reserve or in the evaluation of polycystic ovarian disease (PCOS). AMH levels in PCOS range from 5 ng/mL to several fold higher than that, with much overlap in the ranges seen with GCT [7].

On magnetic resonance imaging (MRI), these tumors are typically described as solid masses with a variable amount of cystic component. GCT exhibit varied histologic patterns which can be mixed in a single specimen. The protean histologic patterns can create perplexing sonographic manifestations [8]. The ultrasound appearance sometimes shows a homogeneous isoechogenic ovoid ovarian mass, similar in appearance to an ovarian endometrioma [8].

## Case report

**Fig. 1:**
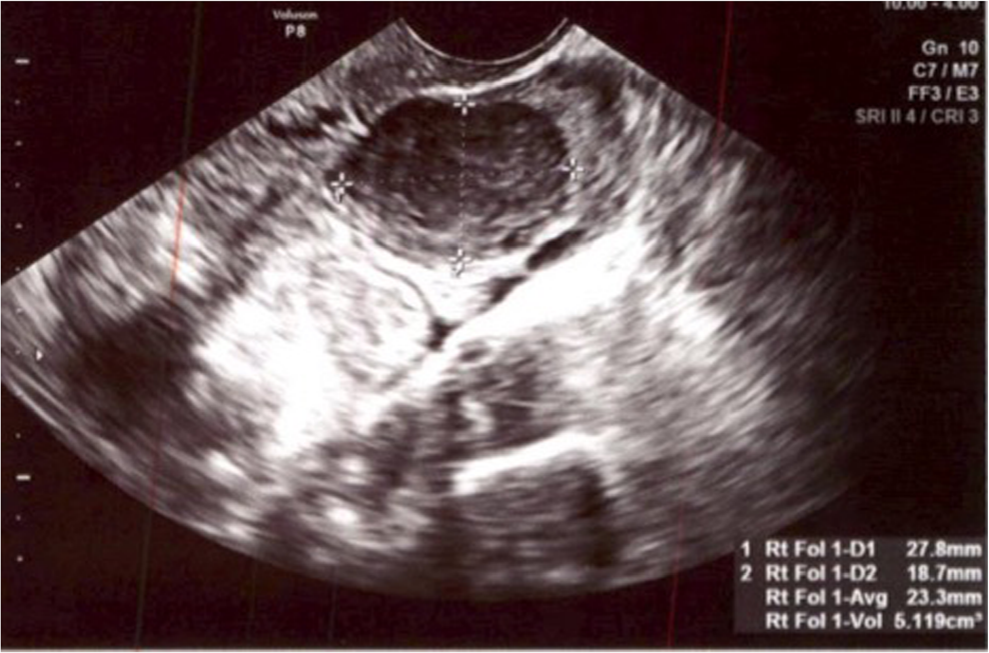
Case 1. Right ovarian mass presumed to be an endometrioma during ultrasound at the patient’s initial presentation. This homogeneous relatively uniform ovoid mass was initially thought to represent an ovarian endometrioma, instead of the granulosa cell tumor it proved to be

A 37-year-old gravida 0 Asian female presented for elective fertility preservation, as she and her partner wanted to focus on their careers. Past medical, surgical, and family histories were unremarkable. She experienced menarche at age 12 and had irregular menstrual cycles at q34–43 day interval. The partner was healthy with a normal semen analysis. Infertility evaluation was remarkable for an elevated AMH of 34 ng/mL, normal FSH of 4.8, and LH of 7.4 IU/mL. The patient underwent a transvaginal ultrasound which revealed a right ovarian mass measuring 2.5 cm that was suggestive of an endometrioma (Fig. 1). Left ovary appeared normal. Both ovaries had antral follicle count of 8 each. The hysterosalpingogram revealed normal cavity and bilateral patent tubes.

Almost 2 years later, upon deciding to proceed with IVF and embryo freezing, the patient had a baseline scan before starting IVF. The left ovary contained four antral follicles and a small irregular cyst thought to be a corpus luteum. Ultrasound of the right ovary revealed six antral follicles and a cystic and solid 3.4-cm mass that was suspicious for a granulosa cell tumor. This possibility was discussed with the patient. Given the uncertainty of bilateral involvement, stimulation was begun while simultaneously consulting with gynecologic oncologists. The patient was started on gonadotropin stimulation with the GnRH-antagonist protocol and rFSH 225 IU daily. Further ultrasound examination led to the conclusion of right-sided GCT and resolving left ovarian corpus luteum. Inhibin B was > 1300 pg/mL on day 6 of stimulation, with the rest of the tumor markers, LDH, AFP, CEA, CA19-9 and CA 125, within normal limits. AMH was 36.2 ng/mL. The Gynecology Oncology team agreed that it was reasonable to proceed with IVF egg retrieval and to avoid entering the affected ovary. The patient was counseled regarding concern for malignancy and the risk of seeding of malignant cells if both ovaries happen to be affected. The patient was aware of risk of bilateral oophorectomy during surgical staging should MRI reveal bilateral ovarian disease, and losing fertility potential. The lesion grew from 3.8 × 3.0 cm at baseline to 5.0 × 4.0 cm at the completion of ovarian stimulation. The right ovary developed four follicles measuring 19, 19, 19, and 17 mm mean diameter. The left ovary had follicles measuring 20, 19, 17, and 13 mm mean diameter. Ovarian maturation was triggered with leuprolide after a total rFSH dose of 1825 IU, and a peak serum estradiol reached 2070 pg/mL. She had five oocytes retrieved from the left ovary and three blastocysts cryopreserved. The right ovary was left undisturbed.

Post-retrieval MRI revealed a 3.8 × 4.5 cm solid mass in the right ovary, with the left ovary appearing normal, status post oocyte retrieval. Tumor volume increased by 78% between the baseline ultrasound and MRI, presumably in response to gonadotropin stimulation. Shortly thereafter, she underwent a laparoscopic right salpingo-oophorectomy, pelvic washings, dilation, and curettage and was discharged home the same day. Pathology revealed stage 1A adult-type granulosa cell tumor with lymphovascular space invasion and scattered theca cell without atypia or mitotic activity. Post-operatively, inhibin B fell to 119 pg/mL. The patient will be followed at q6-month intervals with physical exam and tumor markers as per NCCN guidelines.

### Case 2

A 38-year-old gravida 1 para 0010 female presented with infertility of 15 years duration. The patient was initially referred 9 years earlier by her gynecologist and underwent a laparoscopy and hysteroscopy with findings of stage I endometriosis, patent right fallopian tube, and intrauterine synechia. The patient’s obstetrical history was significant for a left cornual ectopic pregnancy, for which she underwent an exploratory laparotomy with left cornual resection 16 years ago. She had irregular menses since menarche at age 13 years old and typically has spotting for about 5 days every other month. The patient had no allergies and was not on any medications. On initial evaluation, she underwent a transvaginal ultrasound which was significant for a 7 × 5 cm solid homogeneous appearing ovoid mass of her left ovary, which was suspicious for a granulosa cell tumor. The patient had an elevated baseline AMH of 14.3 and normal CA-125 of 13. Referral to Gynecology Oncology resulted in a laparoscopic left salpingo-oophorectomy and lysis of adhesions without complications. Surgical pathology revealed a granulosa cell tumor. Post-operatively, repeat AMH fell to 0.64 ng/mL, and inhibin B to 14 pg/mL. Six months later, the patient underwent ovarian stimulation for IVF and conceived an intra-uterine pregnancy, which unfortunately ended in a first trimester loss. Further infertility treatment is ongoing.

## Discussion

We present two cases of adult granulosa cell tumor in patients desiring fertility. This is the first case report of using IVF therapy in patients with granulosa cell cancer. In case 1, the granulosa cell tumor was not suspected until after ovarian stimulation had begun. In retrospect, this could have been suspected sooner. The patient was mistakenly presumed to have PCOS because of her menstrual irregularity and high AMH. Hirsutism in Asian women with PCOS is sometimes less pronounced [9]. The ovary containing the tumor and producing high levels of AMH responded to ovarian stimulation in a similar way to the unaffected contralateral ovary. Surgical therapy and staging occurred after three blastocysts were cryopreserved, allowing some chance of future fertility. There was nearly 80% growth of tumor volume in response to gonadotropin stimulation during IVF. We do not know of the effect of stimulation on the risk of tumor recurrence. In the second case, the GCT had a more common ultrasound appearance, was confirmed with laboratory testing, and was treated before proceeding with IVF.

In summary, granulosa cell tumor can be confused with ovarian endometrioma on transvaginal ultrasound as GCT has a wide variety of appearances. When an ovarian mass is visualized, serum AMH elevation should raise the suspicion of GCT. Significant AMH elevations in a patient without the stigmata of PCOS should also raise the suspicion of GCT. MRI and serum for inhibin B should be conducted for confirmation. These tumors must be removed intact, without capsular rupture to avoid the need for platinum-based chemotherapy. Unilateral, simple oophorectomy allows the opportunity for future fertility. Despite high levels of local AMH production, ovaries containing GCT respond to gonadotropin stimulation like the contralateral ovary with intra-ovarian tumor volume increasing during gonadotropin therapy.
